# Evidence for scale up: the differentiated care research agenda

**DOI:** 10.7448/IAS.20.5.22024

**Published:** 2017-07-21

**Authors:** Anna Grimsrud, Ruanne V. Barnabas, Peter Ehrenkranz, Nathan Ford

**Affiliations:** ^a^ HIV Programmes & Advocacy, International AIDS Society, Cape Town, South Africa; ^b^ Departments of Global Health and Medicine, University of Washington, Seattle, USA; ^c^ Department of Global Development, Bill & Melinda Gates Foundation, Seattle, WA, USA; ^d^ Department of HIV/AIDS & Global Hepatitis Programme, World Health Organization, Geneva, Switzerland

**Keywords:** Differentiated care, differentiated service delivery, models of care, client-centred, health systems, antiretroviral therapy, research agenda

“*It’s not about everybody getting the same thing. It’s about everybody getting what they need in order to improve the quality of their situation.” Cynthia Silvia Parker, Interaction Institute for Social Change.*

The scale up of antiretroviral therapy (ART) to more than 18 million people living with HIV (PLHIV) [1], primarily in low- and middle-income countries, is one of history’s greatest public health achievements. Life expectancy among PLHIV on ART has improved worldwide [2], and in some settings hardest hit by the epidemic, rapid gains have been seen. For example, in KwaZulu-Natal, South Africa, life expectancy has increased three times faster than the previous highest recorded increase – seen in Japan as it recovered from World War II [3]. Advances in treatment over the past decade combined with increased access to care have transformed HIV into a manageable, chronic disease [4]. HIV has led the way in providing a platform that could lead to the successful management of chronic diseases in resource-limited settings [5,6].

We now live in an era where it is recommended that all PLHIV initiate ART as soon as possible following diagnosis [7], and consequently the size of the potential treatment cohort has almost doubled, from 18.2 million to 36.7 million [1,8]. However, global funding for the epidemic remains flat or declining in present-day value [9,10]. With the cost of antiretroviral drugs having decreased 100-fold in the past decade [11], further cost reductions are dependent on making “efficiency gains” within the healthcare system. However, what about the client – the person living with HIV? While there is evidence of high rates of attrition after starting ART [12], among those who do stay on treatment, high levels of viral suppression are achieved [13,14]. Yet, as the world pushes towards 90–90–90 targets, the resources being utilized to achieve the current successes will be further stretched and known challenges, like rates of attrition, may be exacerbated.

Business as usual will not be enough to meet global treatment goals. However, much of the success of ART scale up to date has been attributed to its simple, one-size-fits-all approach [15] in which most PLHIV receive facility-based care from professional healthcare cadres at frequent intervals regardless of their context, clinical characteristics or subpopulation. Differentiated care or differentiated service delivery is defined as “a client-centred approach that simplifies and adapts HIV services across the cascade, in ways that both serve the needs of PLHIV better and reduce unnecessary burdens on the health system” [16]; it is an attempt to maintain a public health approach while acknowledging that people’s needs change over time.

The majority of published evidence of differentiated care has been limited to ART delivery for stable adults in high-prevalence settings in sub-Saharan Africa. Largely pioneered by the medical aid agency Médecins Sans Frontières (MSF), four innovative service delivery models have emerged in response to context-specific client needs and health systems challenges met in different countries [17]:

- **Client-managed groups** (known as community adherence groups or CAGs) in Mozambique to address the limitation of a supply chain that could only provide one month of ART refills at a time to PLHIV who lived far from the facilities [18–20]. This model has been researched in Haiti [21], Lesotho [22], Malawi, South Africa and Zambia with implementation in Uganda (as community client-led ART delivery or CCLAD) and national policy support for scale up in Kenya, Swaziland [23] and Zimbabwe.
- **Healthcare worker-managed groups** (known as adherence clubs) in South Africa to address high client volumes and long wait times [24,25]. This model has national policy support for scale up in Kenya [26] (as facility-based distribution groups), Swaziland [23] (as facility-based treatment clubs) and Zimbabwe (as club refills). Pilot implementation is ongoing in the Democratic Republic of the Congo (DRC) and Zambia (as adherence groups).
- **Facility-based individual models** (known as the six-monthly appointment or SMA programme) in Malawi to address long wait times at facilities in a context where three-monthly ART refills were included in national guidelines, and six-monthly clinical visits could be piloted [27,28]. This model has been piloted or implemented in at least six other countries (Ethiopia, Rwanda, South Africa, Swaziland, Uganda [29] and Zimbabwe) and is commonly referred to as fast-track or multi-month prescription/scripting.
- **Out-of-facility individual models** (known as points de distribution communautaires or PODIs) in the DRC to reduce client costs of transport to clinics and fees for clinic visits [17,30]. Out-of-facility individual models include: mobile outreach (being implemented in Namibia and Swaziland [23]); community drug distributions points (CDDPs) as seen in Uganda [31,32]); the central chronic medicine dispensing and distribution (CCMDD) programme in South Africa; and home-delivery.

These four basic models are proposed as a common nomenclature for describing differentiated care for stable ART clients.

For this special issue of the *Journal of the International AIDS Society*, we issued a global call for abstracts on differentiated care with the aim of deepening the understanding of these approaches and broadening the knowledge base. In this editorial, we present 10 priority areas for investigation, highlighting areas where manuscripts in the supplement have made contributions and noting gaps that remain. These priority research areas are informed by an appraisal of the literature and discussions with representatives from networks of people living with HIV, ministries of health, implementing partners, researchers, normative agencies and funders.

## 1. Beyond the context of sub-Saharan Africa and high-prevalence settings

Two articles by MSF highlight examples of differentiated ART delivery outside of sub-Saharan Africa. Mesic et al. report on the client and health system implications of an individual facility-based model for stable clients in Yangon, Myanmar [33]. Their findings present novel evidence of the success of differentiated ART delivery for stable clients in a concentrated HIV epidemic. Work from South Sudan, the Central African Republic and the DRC is shared in the article by Ssonko et al., differentiating ART services both for ART initiation and delivery, were implemented to support client outcomes in challenging environments [34]. Finally, a commentary by Nsanzimana et al. on the phased implementation of an individual facility-based model in the Rwandan national HIV programme emphasizes the relevance of differentiated ART delivery in Rwanda [35], a low-prevalence HIV setting [36,37].

With the exception of limited pilot data from Haiti [21], the three articles in this supplement are the first to assess how differentiated care can be advantageous in different contexts. What is the role and the impact of differentiated care in contexts of low prevalence and low coverage?

## 2. Beyond stable adults: impact for key and vulnerable populations

Differentiated care does not only mean ART delivery for stable clients, despite the majority of the evidence coming from this approach. Potentially, those who stand to benefit the most from a differentiated care approach are the key and vulnerable populations who do not access routine clinic care and may require more attention to achieve quality HIV care outcomes.

Macdonald and colleagues make a strong argument for the inclusion of key populations – men who have sex with men, transgender people, people who use drugs and persons in closed settings – within differentiated care [38]. Limited data on ART outcomes among key populations are available, with the majority of funding and programming for this group targeted at the front end of the HIV care cascade (i.e. HIV testing and prevention). Given the structural barriers, including criminalization and stigma that adversely impact key populations from accessing and receiving quality care, the question of whether differentiated care can mitigate these barriers is raised.

As “treat all” is being implemented globally, there are a number of lessons to be learned from the implementation of Option B+ for pregnant women. A large body of evidence highlights the poor rates of retention among women who are initiated onto ART during pregnancy. In the article by Myer et al, the six-month post-partum outcomes of women who are initiated during pregnancy are reported after their self-selection into a healthcare worker-managed group (community adherence clubs) or referral to their local primary care clinic [39]. While the study is limited in reporting of outcomes (the study could not assess if women referred to but not retained in adherence clubs were retained on other ART services), it draws attention to the reality that PLHIV may benefit from different service delivery models throughout their treatment lifetime.

Clearly, more research for both pregnant women and key populations is required – as is more work focusing on children, adolescents and young people, men and other marginalized populations. Can differentiated care engage and retain those populations who are currently not in the clinic-based HIV care system?

## 3. Beyond pilots: overall impact

Public health authorities often seek evidence that new models of care have proved to be feasible, acceptable, cost efficient and effective outside of research settings; yet such “real-life” evaluations of programmes that have achieved partial or full scale are rare. The programme evaluation of 30 high-volume facilities in Malawi providing different models of care alongside traditional models by Prust et al. was a response to a ministry request for an independent assessment of ongoing programmes [40]. It augmented routinely collected clinical data with data on health systems costs, as well as PLHIV and healthcare provider experience, to provide the policymakers with the evidence to guide policy decisions.

Tsondai et al. assessed outcomes of clients in the healthcare worker-managed adherence clubs in the Western Cape of South Africa, with a random sample of 10% of clients from non-research supported sites [41]. With high rates of retention and viral suppression, this evidence confirms that good outcomes among patients differentiated into a healthcare worker-managed groups are not limited to pilot projects.

Additional programme evaluations, cohort studies, step wedge or other smart implementation science designs are needed to bolster evidence for the benefits of differentiated care at scale. Outcomes measures should include retention in care, viral suppression, data on patient and healthcare worker experience, healthcare worker productivity, as well as health system and patient costs. What will be the “real-life” outcomes when models are scaled, such as the streamlined delivery model presented by Kwarisiima et al. [42]? What is the best strategic mix of models to achieve optimal outcomes in a given setting?

## 4. Cost – for the health system and also for clients

While the primary purpose of differentiated care is to improve patient outcomes and not to save costs, donor funding for HIV programmes is declining. Costing of these models is therefore important to inform choice by national governments and advocate for donor support for scale up. Such costing work is complicated and should be expanded to include the health systems costs inclusive of training and implementation and the client costs (e.g. transportation, missed opportunity/work).

Implementation of differentiated care approaches is facilitated by access to viral load monitoring, and previous modelling suggests that the costs of viral load monitoring can be offset by taking action on results to refer clients with high viral loads to intensified clinical care or shift the engagement of those with suppressed viral load to a reduced frequency of visits [43]. In the article noted above, Prust et al. describe evidence of such costs among three different models of care in Malawi [40].

Adding to previously published evidence on cost [19,29,32,44–48], Barker et al. suggest that differentiated care models could decrease health systems costs in 38 countries in sub-Saharan Africa [49]. While some costing work has been done, an additional benefit of costing may be identifying areas for efficiencies. What are the clients’ costs (including time spent waiting or in transit) when engaged in a differentiated model of care vs. a traditional model? Which models lead to the greatest increase in health care worker productivity and make the best use of existing infrastructure?

## 5. Differentiated care across the cascade

The concept of differentiated care is applicable across the care cascade from prevention to testing to viral suppression. While the majority of studies in this issue are limited to the provision of ART, an article from Uganda by Asiimwe et al. provides evidence that lay cadres can be leveraged to expand testing services and supporting linkage of PLHIV to care [50].

Many unanswered questions remain with respect to how to improve outcomes at the front end of the cascade. What service delivery models can be used to improve testing, linkage and initiation of ART? And can we leverage the models of differentiated ART delivery to support this? For example, can we use community-based client-managed group members to increase rates of testing and linkage [51,52]? Can ART initiation be included within models of community testing? And with regards to prevention, what service delivery models will work to improve access, uptake and adherence to pre-exposure prophylaxis (PrEP)?

## 6. Evidence for extending refills

The review from Apollo et al. summarizes the evidence base used by the World Health Organization to make its 2016 recommendations of 3–6 monthly clinical visits and 3–6 monthly ART refills for stable clients [7,53]. Can these intervals be widened, and do they apply to all populations? In South Africa, clinical consultations for stable clients on ART are annual and so analyzing national routine data may support extending the frequency of clinic visits. Further, for children who are 2–5 years of age, the misconception of frequent dosing changes (which mainly occur in the first year) has led to an insistence on frequent clinic visits. And for adolescents, there is desire for frequent visits and contact given their high rates of attrition, despite the evidence that frequent clinic visits leads to higher rates of loss to care among adults. Similarly, there is an assumption that someone who appears to be non-adherent requires additional contact with the health system. Can we define which segments of people – shift workers, students in a full day of school – are most likely to experience frequent visits as a barrier to adherence?

The remaining four research priorities are not addressed within this special issue, and this may reflect an overall lack of evidence and experience in these domains. These include client and healthcare worker preference, integration of co-interventions, integration of care for co-morbidities and co-infections, and assessing the impact of differentiated care for clinically unstable patients (e.g. clients with high viral load, uncontrolled comorbidity, etc.).

## 7. Client choice, satisfaction and quality

If the client is at the centre, then the client’s voice must be central to the design of differentiated care. When assessing which model(s) to choose, it is fundamental to speak with the clients or recipients of care [54]. Further, for differentiated care to become integrated within healthcare systems, there will also need to be perceived benefits for healthcare workers. Is there a correlation between implementation of differentiated care, decreased costs, improved quality and improvements in client and healthcare worker experience?

## 8. Integration of co-interventions within HIV differentiated care

HIV services cannot operate as vertical programmes. If differentiated care is to be successful, models will need to integrate co-interventions. How can HIV self-testing, PrEP isoniazid preventive therapy, cotrimoxazole and other prophylaxis plus simple diagnostic tests (e.g. Lipoarabinomannan or LAM assays to detect TB), be integrated into service delivery models?

## 9. Integration of care for co-morbidities and co-infections within HIV differentiated care

The design of service delivery models for PLHIV must also address co-morbidities and co-infections [26]. With an aging population of PLHIV and a burgeoning epidemic of chronic diseases, services must incorporate treatment and prevention for non-communicable diseases. In addition, integration of tuberculosis services, provision of family planning and availability of opioid substitution therapy are all necessary to realize a client-centred approach. Evidence is needed on the leading causes of morbidity and mortality among HIV-positive persons on ART to indicate which services to integrate with HIV care. How can co-morbidities be integrated with HIV differentiated care?

## 10. Impact of differentiated care for clinically unstable clients

Can clients with high viral loads benefit from being included in differentiated ART delivery models? For example, can community delivery of ART to those most at risk of defaulting lead to improved outcomes? Does the implementation of differentiated ART delivery for stable clients lead to an efficient refocusing of clinic resources towards improved outcomes for those who are unstable? And how can we establish and strengthen community referral to ensure that clients who need intensified clinical support are accessing services in a timely manner?

## Conclusions

The evidence base to support differentiated care is robust and expanding, but many questions remain. Overarching these priority areas for research is the engagement of local policymakers, implementers and PLHIV in the development of a prioritization plan, the design of the studies and the full dissemination of results to support demand creation for differentiated care services within their communities and among healthcare workers. Differentiated care has the potential to be a game changer in the treat all era [55], but will only realize its full potential with scaled implementation and ongoing adaptation informed by implementation research.
